# Efficacy of oseltamivir compared with zanamivir in COPD patients with seasonal influenza virus infection: a randomized controlled trial

**DOI:** 10.1590/1414-431X20209542

**Published:** 2020-11-27

**Authors:** Min Li, Guang-chao Han, Yang Chen, Wen-xiu Du, Fang Liu, Yu-min Chi, Jun-feng Du

**Affiliations:** Section 1, Department of Respiratory and Critical Care Medicine, Cangzhou Central Hospital, Cangzhou, Hebei, China

**Keywords:** Chronic obstructive pulmonary disease, Influenza virus, Oseltamivir, Zanamivir, Randomized controlled trial

## Abstract

Influenza viruses exacerbate chronic obstructive pulmonary disease (COPD) with considerable morbidity and mortality. Zanamivir and oseltamivir are effective in treating influenza. However, their efficacy in relieving influenza symptoms in COPD patients remains unknown, with the lack of controlled trials in this subject. Therefore, we conducted this randomized controlled trial to investigate the clinical efficacy of both interventions in this population. Patients were allocated to two groups (80 patients each): oseltamivir (OSELTA) and zanamivir (ZANA) groups. Oseltamivir (75 mg) was orally administered twice daily for 5 days, while zanamivir (10 mg) was inhaled twice daily for 5 days. Clinical parameters including body temperature, influenza symptoms (i.e., sore throat, cough, etc.), and serial blood tests were recorded on days 1, 3, and 7. We analyzed primary (changes in body temperature) and secondary outcomes (changes in non-specific symptoms) using the pre-protocol and intention-to-treat analyses. Differences between groups were assessed using *t*-test. Oseltamivir and zanamivir significantly reduced body temperature on the 3rd day after treatment; however, the number of patients who reported clinical improvement in influenza-like symptoms was significantly higher in the OSELTA group compared to the ZANA group on days 3 (85 *vs* 68.8%, P=0.015) and 7 (97.5 *vs* 83.8%, P=0.003). However, no significant changes in hematological (white blood cells and its subtypes) and inflammatory (C-reactive protein) parameters were noted (P>0.05). Our results suggested that oseltamivir and zanamivir are effective in reducing body temperature, while oseltamivir led to better clinical improvement regarding influenza-like symptoms in patients with COPD.

## Introduction

Chronic obstructive pulmonary disease (COPD) is a common respiratory disease characterized by chronic bronchitis and airflow obstruction. It is an irreversible disease with progressive deterioration of respiratory symptoms (1,2). Persistent airway inflammation is a cardinal feature of COPD (3), which is aggravated by the influence of lifestyle factors, especially smoking (4). Inflammatory cells such as macrophages and neutrophils aggregate in large numbers in the airway mucosa. In addition, there is an increase in bronchial mucosal secretory activity, inflammatory cytokines, and chemokines. People with chronic inflammatory diseases of the respiratory system, such as COPD, have a high risk for influenza complications, because influenza may aggravate the underlying condition or make these individuals susceptible to secondary bacterial infections and pneumonia.

Acute exacerbation of chronic obstructive pulmonary disease (AECOPD) is the main cause of high hospitalization rates and mortality in patients with COPD (5). Respiratory virus infections such as rhinovirus, influenza virus, and respiratory syncytial virus are the main cause of AECOPD, which is triggered by persistent inflammatory response of the respiratory tract induced by virus infection (6). According to previous research, the infection rate of influenza A and influenza B is as high as 28% among AECOPD patients. Therefore, in order to prevent AECOPD, COPD patients with influenza virus infection must be treated timely and effectively. These patients should be vaccinated against influenza each winter and treated with antiviral drugs once infected (7,8). In 2018, a meta-analysis reported that influenza vaccination could be used as an effective preventive measure in patients with COPD; vaccination significantly reduced the frequency of exacerbations compared to placebo (6). However, these findings are based on only two trials with an overall high risk of bias. Until more clinical trials with larger sample sizes are conducted, the use of antiviral drugs in this population is indispensable, particularly in elderly patients and those with a compromised immune system.

Oseltamivir phosphate, a neuraminidase inhibitor, is currently recommended as an effective drug for the treatment of influenza (9,10). Many studies show that both oseltamivir and zanamivir are effective for influenza patients with respiratory complications such as COPD and asthma (11,12). However, there are currently no randomized controlled trials comparing the efficacy of both interventions in treating influenza patients with COPD. Therefore, the aim of this study was to evaluate and compare the clinical efficacy of oseltamivir and zanamivir in COPD patients infected with influenza.

## Material and Methods

### Sample size calculation and random grouping

Since zanamivir is an inhaled preparation, some studies have shown that its compliance is about 90% (13 [Bibr B14]). Therefore, we assumed that the effective rate of zanamivir was 70%, while the anti-virus effect of oseltamivir was 90%. The confidence interval was 95%, and the shedding rate was 20%. According to the proportion of 1:1, this study needed at least 71 cases in the oseltamivir group and zanamivir group. Finally, we included in this trial a total of 160 patients, who met our eligibility criteria and were randomized to receive one of the studied interventions using a random number sequence generator.

### Study population

This study was conducted at Cangzhou Central Hospital, Hebei Province, China, during the period from December 2017 to April 2019. Patients >18 years of age with COPD and influenza were considered eligible to participate in our clinical trial. COPD patients with influenza-like illness of ≤36 h were recruited. Influenza was defined clinically by the presence of fever (axillary temperature of >38°C) along with at least two of the following manifestations: sore throat, cough, headache, muscle or joint aches, and pain. Patients with known or suspected hypersensitivity, as well as patients with severe intolerance to any of the administered interventions, were excluded. Other reasons for exclusion were: impaired respiratory function, history of congestive heart failure, uncontrolled/poorly-controlled diabetes, history of immunosuppressive therapy (immunosuppressant, antineoplastic drugs, etc.), or immunocompromised state (i.e., AIDS), renal dysfunction (estimated creatinine clearance rate of 50 mL/min), ischemic heart disease or severe arrhythmia, corrected QT interval (QTC) of 480 ms, bradycardia (heart rate of 40 beats/min), recent use of influenza antiviral therapy, and suspected bacterial respiratory infection with antimicrobial medication.

### Diagnostic criteria

COPD was defined as a documented history of chronic airflow restriction diagnosed by a healthcare professional or a forced expiratory volume in one second (FEV_1_) of ≤80% of that predicted for age, gender, and height. COPD was diagnosed based on the guidelines proposed by the Global Initiative for Chronic Obstructive Lung Disease (GOLD) in 2020 [14), which is based on the severity of airflow limitation, symptoms assessment (using the COPD assessment test (CAT) or the modified Medical Research Council (mMRC) questionnaire), and the history of acute exacerbations. All COPD patients were classified as either A (CAT score <10; mMRC 0-1) or B (CAT score ≥10; mMRC ≥2), with a history of fewer acute exacerbations. The diagnosis of influenza was confirmed (day 1) by viral culture (through nasopharyngeal swabs) and polymerase chain reaction (PCR). COPD patients with positive influenza virus infection A or B were then divided into two groups based on the administered intervention.

### Ethical considerations

This study was approved by the institutional review board (IRB) of Cangzhou Central Hospital, Cangzhou, Heibei, China (2017-047-01). Prior to conducting the trial, written informed consent was obtained from each patient willing to participate after being given a detailed explanation about the trial.

### Intervention measures

Patients were randomly divided into two groups: oseltamivir group (OSELTA) and zanamivir group (ZANA). The OSELTA group (80 patients) was treated with orally-administered oseltamivir (Shanghai Roche Pharmaceutical Co., Ltd., H20090377), 75 mg twice a day for 5 days. The ZANA group (80 patients) was treated with inhaled zanamivir (GlaxoSmithKline Australia Pty Ltd., H20090553), 10 mg twice a day for 5 days. In addition, patients with dyspnea, chest tightness, or shortness of breath were given oxygen ventilation. At baseline, patients in both groups were supplied with relief medications in the form of paracetamol (acetaminophen) and dextromethorphan (pholcodine); however, they were advised to avoid taking these drugs unless necessitated by the severity of their symptoms.

### Observation index

The primary outcome of our trial was a difference in body temperature from baseline in each group. Secondary outcomes were related to changes in influenza symptoms and some laboratory parameters (white blood cell count and differentiation). On the 1st, 3rd, and 7th days, body temperature and influenza-like symptoms, such as fever, nasal congestion/runny nose, sore throat, cough, myalgia, weakness, headache, and shiver, were reported by patients in both groups. Laboratory blood investigations in the form of total leucocyte count (10^9^/L), neutrophil count (×10^9^/L) and percentage, and lymphocyte count (×10^9^/L) and percentage were also recorded at baseline and at day 7 after treatment to monitor post-treatment changes in these laboratory parameters.

### Efficacy analysis

Normalized body temperature was defined as 37°C. Clinical improvement of influenza-like symptoms was defined when presenting symptoms were recorded as ‘none' or ‘mild' compared to initial presentation, and maintained normal for at least 24 h. These criteria have been previously described in the literature (11). Comparisons were made regarding changes in body temperature, the time taken for symptom relief, and the time taken for the body temperature to return to normal. The efficacy criteria included three items: 1) not effective: after 72 h of treatment, the patient still had fever and the symptoms of physical discomfort were aggravated or not relieved; 2) effective: the patient's body temperature gradually recovered within 72 h of treatment and the symptoms of physical discomfort improved or partially disappeared; and 3) markedly effective: patients' temperature returned to normal, and the symptoms of physical discomfort improved significantly within 48 h from intervention. Total efficiency was calculated as (effective + markedly effective)/total number of cases ×100. Recorded adverse reactions included nausea, abdominal pain, vomiting, and diarrhea.

### Statistical analysis

Primary and secondary outcomes were evaluated using the pre-protocol and the intention-to-treat (ITT) analysis principles, respectively. Missing data in the ITT analysis were computed using the multiple interpolation methods. All statistical analyses were performed using the Statistical Package for Social Science (SPSS) software, version 2.0 (IBM, USA). Categorical variables are reported as numbers and percentages, while continuous variables are reported as means±SD. Statistical differences among groups were determined by Student's *t*-test. For binary categories, we used the chi-squared test. A follow-up analysis was conducted using the least significant difference (LSD) test. A P-value of <0.05 was considered the cut-off value of statistical significance.

## Results

### Patient demographics and baseline characteristics

None of the 160 patients dropped-out from the study, had a severe allergic reaction to the administered interventions, were lost to follow-up, or violated the study protocol. One hundred percent of the patients recruited in each study group were included in the final analysis.

In the ZANA group, there were 44 (55%) females and 36 (45%) males with an average age of 56.0±5.9 years. The disease course (influenza) ranged from 1 to 7 days prior to drug administration, with 44 patients being infected with influenza A and 36 with influenza B virus. In the OSELTA group, there were 32 (40%) females and 48 (60%) males, with an average age of 55.4±6.6 years. The disease course ranged from 1 to 4 days prior to drug administration, with 42 patients being infected with influenza A and 38 with influenza B virus. At baseline, there were no significant differences regarding gender, age, classification ratio of virus infection, and lung function (FEV1) between the groups (P>0.05) (Table 1). Moreover, both groups had similar mean CAT scores at baseline, indicating that COPD patients in both groups had similar severity at baseline.

**Table 1:** Demographic characteristics of the chronic obstructive pulmonary disease patients with influenza virus infection who received oral oseltamivir (OSELTA group) or inhaled zanamivir (ZANA group).

Variable	OSELTA group (N=80)	ZANA group (N=80)	Statistics
Age (years), mean ± SD	55.4±6.6	56.0±5.9	*t*=0.517; P=0.060
Gender, n (%)			
Male	48 (60%)	36 (45%)	χ^2^=3.609; P=0.057
Female	32 (40%)	44 (55%)	χ^2^=3.609; P=0.057
Nasal swab test, n (%)			
Influenza A	42 (52.5%)	44 (55.0%)	χ^2^=0.101; P=0.751
Influenza B	38 (47.5%)	36 (45.0%)	χ^2^=0.101; P=0.751
CAT, mean ± SD	10.2±4.6	10.7±3.9	*t*=1.032; P=0.301
FEV_1_%, mean ± SD	76.7±3.9	76.0±3.5	*t*=1.255; P=0.211

χ^2^: chi-squared test; *t*: Student’s *t*-test; CAT: COPD assessment test; FEV1: functional end-expiratory volume at 1 second.

Most patients presented with typical influenza symptoms and non-specific respiratory symptoms were predominant. Patients in the OSELTA group complained of moderate to severe fever (96.3%), stuffy nose/nasal discharge (53.8%), weakness (51.3%), headache (47.5%), and myalgia (46.3%). Similarly, patients in the ZANA group complained of moderate to severe fever (97.5%), stuffy nose/nasal discharge (57.5%), weakness (52.5%), headache (55.5%), sore throat (53.5%), and myalgia (45.0%). The respiratory symptoms score did not differ significantly between studied groups (P>0.05).

### Temperature comparison by ITT analysis

At baseline (day 1), patients in both groups had similar mean body temperature (P=0.882). On day 3, both oseltamivir and zanamivir were effective in normalizing body temperature, even though the reduction in body temperature was more pronounced in the OSELTA group (P=0.009). Noteworthy, the reduction in body temperature was similar between the OSELTA and ZANA groups on the 7th post-treatment day (P=0.360) (Table 2). A higher percentage of patients in the OSELTA group had normalized body temperature at days 3 and 7 after treatment compared to the ZANA group, respectively. However, this difference did not reach statistical significance (P>0.05) (Table 3). Furthermore, the time taken for the body temperature to return to normal did not differ between studied groups (P=0.153).

**Table 2:** Changes in body temperature (°C) on days 1, 3, and 7 of treatment of chronic obstructive pulmonary disease patients with influenza virus infection treated with oral oseltamivir (OSELTA group) or inhaled zanamivir (ZANA group).

Assessment day	OSELTA group (N=80)	ZANA group (N=80)	Statistics
Day 1			
	38.2±0.8	38.2±0.7	Δ°=0.02; 95%CI=-0.2049 to 0.2833*t*=0.148; P=0.882
Day 3			
	37.0±0.6	37.3±0.7	Δ°=0.23; 95%CI=-0.4507 to -0.0270*t*=2.631; P=0.009
Day 7			
	36.6±0.2	36.5±0.3	Δ°=-0.04; 95%CI=-0.0193 to 0.1379*t*=-0.918; P=0.360

Data are reported as means±SD. The statistics column shows the results of the Student's *t*-test modified for comparability (non-inferiority) testing. Δ° is the mean difference between the OSELTA group and the ZANA group.

**Table 3:** Comparison of clinical improvement of influenza non-specific symptoms on days 3 and 7 of treatment of chronic obstructive pulmonary disease patients with influenza virus infection treated with oral oseltamivir (OSELTA group) or inhaled zanamivir (ZANA group).

Assessment day	OSELTA group (N=80)	ZANA group (N=80)	Statistics
Day 3			
Body temperature returned to normal	41 (51.3%)	32 (40.0%)	χ^2^=2.041; P=0.153
Improvement in clinical symptoms	68 (85.0%)	55 (68.8%)	χ^2^=5.942; P=0.015
Day 7			
Body temperature returned to normal	80 (100.0%)	77 (96.3%)	χ^2^=3.057; P=0.080
Improvement in clinical symptoms	78 (97.5%)	67(83.8%)	χ^2^=8.901; P=0.003

Data are reported as numbers (percentages). Normal body temperature = 37°C. χ^2^: chi-squared test.

### Comparison of improvement of flu-related non-specific symptoms

The effects of oseltamivir on clinical symptoms of flu were discernible for the 7 days of treatment. On the 3rd day, patients who received oseltamivir were significantly more likely to report clinical improvement of influenza symptoms compared to the ZANA group (P=0.015). On the 7th day, a similar observation was noted where patients who received oseltamivir were significantly more likely to report clinical improvement compared to the ZANA group (P=0.003) (Table 3).

### Comparison of routine blood tests and CRP

Results of white blood cell (WBC) count, neutrophil count, lymphocyte count, and C-reactive protein (CRP) were recorded at baseline (day 1) and day 7 after treatment (Table 4). There were no significant differences in WBC count between the OSELTA group and the ZANA group on day 1 (P=0.331). After treatment (day 7), the WBC count of the two groups was similar for the OSELTA group and the ZANA group (P=0.888). Similarly, no significant differences were noted before and after treatment between both groups as regards lymphocyte and neutrophil percentages as well as CRP (P>0.05).

**Table 4:** Comparison of blood test parameters before and after treatment with oral oseltamivir (OSELTA group) or inhaled zanamivir (ZANA group) of chronic obstructive pulmonary disease patients with influenza virus infection.

Indices	OSELTA group (N=80)	ZANA group (N=80)	Statistics
White blood cells (×10^9^/L)			
Day1	6.3±2.9	5.9±3.0	*t*=-0.976; P=0.331
Day7	6.9±1.8	6.9±1.7	*t*=0.141; P=0.888
Neutrophils (×10^9^/L)			
Day1	3.5±2.0	3.1±2.0	*t*=-1.187; P=0.237
Day7	4.1±1.1	4.1±1.1	*t*=0.140; P=0.889
Neutrophils (%)			
Day1	55.8±19.7	53.7±19.5	*t*=-0.701; P=0.484
Day7	59.5±5.5	59.5±5.7	*t*=-0.052; P=0.959
Lymphocytes (×10^9^/L)			
Day1	2.7±1.9	2.6±1.8	*t*=-0.278; P=0.782
Day7	2.6±0.8	2.7±0.8	*t*=0.311; P=0.756
Lymphocytes (%)			
Day1	41.1±19.8	43.2±19.8	*t*=0.644; P=0.520
Day7	37.6±6.1	38.0±6.2	t=0.410; P=0.682
C-reactive protein (×10^9^/L)			
Day1	59.9±36.6	59.9±32.7	*t*=0.013; P=0.990
Day7	5.3±2.8	5.1±3.0	*t*=-0.466; P=0.642

Data are reported as means±SD. *t*: Student’s *t*-test.

## Discussion

COPD, a heterogeneous disease with global distribution, is characterized by cough, chest distress, dyspnea, and shortness of breath (15,16). Acute exacerbation of COPD often leads to serious symptoms, respiratory failure, and respiratory acidosis. The pathogenic microorganisms that lead to the deterioration of COPD can damage the myocardium (17,18). Influenza virus infection is one of the common causes of acute exacerbation of COPD, with potentially fatal consequences (19,20). Therefore, it is suggested that patients with COPD should receive preventive treatment in the form of influenza vaccine to reduce the frequency of pulmonary complications (21,22). Even though vaccination has shown a significant reduction in the frequency of exacerbation in COPD patients, the level of evidence recommending the vaccination in this population remains low, with a minimal number of clinical trials (6). Moreover, the curative effect of influenza vaccination on the elderly population, as well as patients with underlying pulmonary complications, is significantly lower than that of the healthy population.

Neuraminidase inhibitors (NAIs) exhibit an effective inhibitory activity against influenza virus. The efficacy and safety profiles of zanamivir and oseltamivir with the addition of peramivir, as different forms of NAIs, should be assessed in high-risk patients, especially those with respiratory complications. It has been reported that both zanamivir and oseltamivir are effective adjuvants combined with vaccine for the treatment of influenza patients with asthma and/or COPD (23). However, zanamivir is an inhaled preparation, and its device affects compliance and efficacy, which is worse than that of oral oseltamivir. Furthermore, studies have shown that zanamivir may reduce lung function and induce bronchospasm in patients with asthma and/or COPD (12). Therefore, for patients with potential respiratory diseases, it is recommended to use a rapid and effective inhalation bronchodilator when inhaling zanamivir. Apart from poor compliance, zanamivir may have a potential impact on lung function. Therefore, it is necessary to evaluate and compare the efficacy of oseltamivir and zanamivir for the treatment of influenza patients with COPD.

In this study, we compared the clinical effectiveness of both oseltamivir and zanamivir regarding the resolution of high body temperature and other influenza non-specific symptoms. We noted that both drugs resulted in a significant reduction in body temperature on the third day of treatment. Furthermore, a greater proportion of patients on oseltamivir had normalized body temperature (37°C) on the third day compared to those on zanamivir (51.3 *vs* 40%). Moreover, oseltamivir-treated patients showed significant improvement in clinical symptoms compared to those in the ZANA group. However, there were no significant differences between groups regarding the hematological profile, including WBC, neutrophil count and percentage, lymphocyte count and percentage, and CRP.

Oseltamivir is a safe and effective anti-influenza virus therapy, which is widely used in clinical practice (24). It inhibits the activity of influenza virus neuraminidase and therefore inhibits the progeny virus from budding off host cells (9). Oseltamivir phosphate is the first orally effective neuraminidase inhibitor to be approved by the Food and Drug Administration (FDA) (25). When treatment is initiated promptly, the drug can effectively interfere with viral multiplication. Additionally, oseltamivir has been reported to have a low propensity for drug resistance (26). The existing literature shows that oseltamivir phosphate can significantly shorten the duration of major clinical symptoms and signs in the treatment of influenza, and can have a significant preventive effect on exposure to influenza (27). The results of this study confirmed these literature reports as patients on oseltamivir were significantly more likely to experience clinical improvement (resolution of non-specific influenza symptoms) on days 3 and 7 after treatment compared to zanamivir.

Zanamivir, an influenza virus neuraminidase inhibitor, is clinically used as an inhalation formulation (28). The inhibition of influenza virus occurs in a slow-binding manner and is highly specific. Zanamivir has been shown to be effective and well-tolerated, with only minor harmful effects (except for bronchospasm, which should be carefully investigated). A previous study reported that 67% of healthy adults achieved prevention against influenza virus with fever control (29). The use of zanamivir can significantly reduce the incidence of complications with bronchitis and pneumonia in susceptible people by over 50% and reduce the need for antibiotic treatment by as high as 24% (30–32).

Based on the results of this study, both temperature control and resolution of clinical symptoms achieved by oseltamivir were superior to those observed in the zanamivir-treated group (Table 3). However, both drugs resulted in normalization of body temperature by day 7 after treatment. On the other hand, no significant differences were noted in the various inflammatory indices of hematological examinations between the two groups (Table 4, P>0.05). These observations indicated that oseltamivir had better clinical outcomes compared to zanamivir, but the two drugs showed no significant differences in improving inflammatory parameters. Both drugs reduced body temperature in COPD patients with influenza.

Although our study provides helpful insights into the clinical improvement of influenza virus infection in patients with COPD, we faced some limitations. Firstly, we only analyzed the clinical efficacy of oseltamivir and zanamivir regarding fever and influenza-like symptoms, while the viral titer was not assessed after treatment. Secondly, patients and outcome assessors were not blinded to the received intervention, and thus, our data should be interpreted with caution. Finally, due to the short follow-up period of 7 days, we could not assess whether oseltamivir or zanamivir affects the frequency of exacerbations in the long term. This needs to be investigated in future clinical trials.

In conclusion, oseltamivir phosphate is a promising treatment for influenza virus infection in patients with COPD. However, more robust clinical trials are still warranted to confirm our observations.
